# Quorum Sensing Signaling Molecules Produced by Reference and Emerging Soft-Rot Bacteria (*Dickeya* and *Pectobacterium* spp.)

**DOI:** 10.1371/journal.pone.0035176

**Published:** 2012-04-23

**Authors:** Alexandre Crépin, Corinne Barbey, Amélie Beury-Cirou, Valérie Hélias, Laure Taupin, Sylvie Reverchon, William Nasser, Denis Faure, Alain Dufour, Nicole Orange, Marc Feuilloley, Karin Heurlier, Jean-François Burini, Xavier Latour

**Affiliations:** 1 Laboratoire de Microbiologie Signaux et Microenvironnement (LMSM) - Normandie Université - Université de Rouen EA 4312 - IUT Evreux, Evreux, France; 2 SIPRE Comité Nord Station de Recherche et de Création Variétale, Bretteville du Grand Caux, France; 3 Institut des Sciences du Végétal (ISV) UPR 2355 - CNRS, Gif-sur-Yvette, France; 4 Fédération Nationale des Producteurs de Plants de Pomme de Terre (FN3PT), Paris, France; 5 Institut de Génétique Environnement et Protection des Plantes (IGEPP) UMR 1349 - INRA, Rennes, France; 6 Laboratoire de Biotechnologie et Chimie Marines (LBCM) - Université de Bretagne-Sud EA 3884, Lorient, France; 7 Microbiologie Adaptation et Pathogénie (MAP) UMR 5240 - Université Claude Bernard Lyon 1-INSA-CNRS-Bayer CropScience, Villeurbanne, France; 8 Department of Food Sciences, University of Nottingham, Sutton Bonington, United Kingdom; University of Wisconsin-Milwaukee, United States of America

## Abstract

**Background:**

Several small diffusible molecules are involved in bacterial quorum sensing and virulence. The production of autoinducers-1 and -2, quinolone, indole and γ-amino butyrate signaling molecules was investigated in a set of soft-rot bacteria belonging to six *Dickeya* or *Pectobacterium* species including recent or emerging potato isolates.

**Methodology/Principal Findings:**

Using bacterial biosensors, immunoassay, and chromatographic analysis, we showed that soft-rot bacteria have the common ability to produce transiently during their exponential phase of growth the *N*-3-oxo-hexanoyl- or the *N*-3-oxo-octanoyl-l-homoserine lactones and a molecule of the autoinducer-2 family. *Dickeya* spp. produced in addition the indole-3-acetic acid in tryptophan-rich conditions. All these signaling molecules have been identified for the first time in the novel *Dickeya solani* species. In contrast, quinolone and γ-amino butyrate signals were not identified and the corresponding synthases are not present in the available genomes of soft-rot bacteria. To determine if the variations of signal production according to growth phase could result from expression modifications of the corresponding synthase gene, the respective mRNA levels were estimated by reverse transcriptase-PCR. While the *N*-acyl-homoserine lactone production is systematically correlated to the synthase expression, that of the autoinducer-2 follows the expression of an enzyme upstream in the activated methyl cycle and providing its precursor, rather than the expression of its own synthase.

**Conclusions/Significance:**

Despite sharing the S-adenosylmethionine precursor, no strong link was detected between the production kinetics or metabolic pathways of autoinducers-1 and -2. In contrast, the signaling pathway of autoinducer-2 seems to be switched off by the indole-3-acetic acid pathway under tryptophan control. It therefore appears that the two genera of soft-rot bacteria have similarities but also differences in the mechanisms of communication via the diffusible molecules. Our results designate autoinducer-1 lactones as the main targets for a global biocontrol of soft-rot bacteria communications, including those of emerging isolates.

## Introduction

An important aspect of bacterial communication is based on the release in the microenvironment of small signaling molecules that can diffuse through cell membranes. The most studied model relies on both synthesis and perception of autoinducer-1 (AI-1). AI-1 signaling molecules are *N*-acyl-homoserine lactones (NAHSLs) that serve for cell-to-cell communication called quorum sensing (QS) [1]. Such regulatory systems allow bacteria to sense cell density and containment within a microenvironment according to which they synchronize the functions of the entire population [2]–[5].

Within soft-rot bacteria, the AI-1 communication system is involved in plant virulence with a considerable importance according to the bacterial species. Among them, phytopathogens belonging to *Dickeya* and *Pectobacterium* genera (formerly grouped in the *Erwinia* genus) are responsible for important diseases affecting plant health and compromising the quality of harvested vegetables and other plant products [6]–[10]. In the psychrotolerant *Pectobacterium atrosepticum*, species adapted to temperate regions and specific to the potato host *Solanum tuberosum* L., AI-1 based-QS controls virulence factors involved in a typical stem infection (blackleg) and tuber maceration. Twenty-six percent of the *P. atrosepticum* SCRI1043 genes are under QS control, including genes coding for plant cell wall degrading enzymes and their secretion systems [11]. AI-1 based-QS can also regulate mechanisms involved in the manipulation of plant cell defenses, like harpin synthesis, leading to the hypersensitive response (HR) in non host plant [12]. Generating signaling molecules is therefore a crucial step within plant-*P. atrosepticum* interactions. Indeed, a single mutation of the NAHSL synthase gene or the degradation of signaling molecules before their release in the microenvironment is sufficient to remove all symptoms on potato and to prevent an HR reaction [12], [13]. In *Pectobacterium carotovorum* species, which are found worldwide in a large range of hosts, AI-1 QS controls similar bacterial traits and is necessary for full virulence and antibiotic synthesis [14]–[16]. As a result of these observations, *Pectobacterium* and its AI-1 QS system has become the target for a novel biocontrol approach, which aims at reducing the expression of virulence systems rather than eradicating the pathogen [17]. This biocontrol strategy is based on the biostimulation of NAHSL-degradative microflora using structural analogs of AI-1 signaling molecules [18]. First evaluated on *S. tuberosum* cultures to control blackleg in Western Europe, it could be extended to other crops and diseases [7], [19], [20].

NAHSL-dependent QS appears less important in virulence of other soft-rot bacteria such as *Dickeya dadantii* 3937 and *Dickeya chrysanthemi* EC16 [21], [22], suggesting a possible failure when using the biocontrol method mentioned above [23]. Moreover, data on the environmental distribution of *Pectobacterium* model strains on which signal studies were conducted are often lacking [14], [16], [24], [25]. Furthermore, many recent epidemiologic reports on devastating potato diseases with worldwide distribution, show emergence of new pectinolytic agents, in which communication systems have not yet been identified [26]–[32]. It therefore appears necessary to screen QS systems encountered in a larger panel of strains comprising both type strains from the current taxonomical described species and emergent virulent isolates. The discovery of the autoinducer-2 (AI-2) QS in three *Pectobacterium* strains [33], [34] as well as assumptions regarding the role of signaling molecules synthesized by both plants and *Proteobacteria* [35]–[37] guided our research and has opened up new prospects for biocontrol, provided that such signals are frequently used by pathogenic bacteria. Particular attention will be given to the indole-3-acetic acid (IAA), a compound that harbors all traits of QS signals and whose production can be autoinduced [38]–[39]. Finally, another point that needs to be updated is the possible interconnections between metabolic pathways involved in signals production.

The aims of this work are (*i*) to characterize the nature and production patterns of AI-1, AI-2, quinolone, indole and γ-amino butyric acid (GABA) signaling molecules synthesized by potato soft-rot bacteria, (*ii*) to evaluate their representation within a collection of *Dickeya* and *Pectobacterium* including type and reference strains with a selection of recent isolates belonging to species described on the potato in European growing areas, and (*iii*) to investigate the possible links between the anabolic pathways of signaling molecules by comparing the kinetics of signal generation and the expression of key genes involved in signal syntheses. To our knowledge, this is the first screening evaluating with comparable parameters the potential of a wide range of pectinolytic bacteria to synthesize various signaling molecules.

## Materials and Methods

### Bacterial strains and plasmids

The characteristics of the bacterial sensors and controls used in this work are presented in Table 1. The strains *Agrobacterium tumefaciens* NT1(pZNLR4) and *Escherichia coli* DH5α(pSB401) were used as biosensors for NAHSL detection. Two *Vibrio harveyi* strains, BB170 and BB120, were used as biosensors of the AI-2 signal and as AI-2 producer for the positive control, respectively. *Pseudomonas aeruginosa* H103 was used as a positive control for the detection of 4-hydroxy-2-heptylquinoline (HHQ) and Pseudomonas quinolone signal (PQS) molecules. The characteristics of the soft-rot bacterial panel used in this work are presented in Table 2.

**Table 1:** Bacterial QS signal producers or biosensors, and plasmids.

Strains/Plasmids	Relevant characteristics	Reference
***Agrobacterium tumefaciens***		
NT1(pZNLR4)	NT1 derivative of strain C58, carrying pZLR4 plasmid, NAHSL biosensor, Gm^r^	[43]
***Escherichia coli***		
*E. coli* DH5α	*φ80lacZ ΔM15 Δ(lacZYA-argF) U169 hsdR17 recA1 endA1 thi-1*	[85]
*E. coli* DH5α(pSB401)	Derivative of strain DH5α, carrying pSB401 plasmid, NAHSL biosensor, Tet^r^	[45]
***Pseudomonas aeruginosa***		
*P. aeruginosa* H103	HHQ and PQS producer	[86]
***Vibrio harveyi***		
*V. harveyi* BB120	Wild-type strain, AI-2 producer	[87]
*V. harveyi* BB170	*luxN*::Tn*5*, Dysfunctional AI-2 receptor, AI-2 biosensor	[88]
**Plasmids**		
pZLR4	*traG::lacZ/traR* reporter system, Gm^r^	[43]
pSB401	NAHSL biosensor *luxRI'*::*luxCDABE* fusion; pACYC184 derived, Tet^r^	[45]

Gm^r^ and Tet^r^ indicate resistance to gentamicin and tetracycline, respectively.

NAHSL, N-acyl homoserine lactones; AI-2, Autoinducer-2; HHQ, 4-hydroxy-2-heptylquinoline; PQS, Pseudomonas quinolone signal.

**Table 2:** Virulence traits and origin of potato soft-rot pathogens.

Strain	Host of origin	Year of isolation	Tuber* soft-rot	HR#	Reference or source
***Pectobacterium atrosepticum***					
*P. atrosepticum* CFBP 1526^T^	*Solanum tuberosum*	1957	+	−	CFBP, species type strain
*P. atrosepticum* CFBP 6276	*Solanum tuberosum*	1999	+	+	[40]
*P. atrosepticum* 100T	*Solanum tuberosum*	2003	+	−	[27]
*P. atrosepticum* RNS 08.30.1A	*Solanum tuberosum*	2008	+	−	V. Hélias' collection
***Pectobacterium carotovorum***					
*P. carotovorum* CFBP 2046^T^	*Solanum tuberosum*	1952	+	+	CFBP, species type strain
*P. carotovorum* EC153	*Capsicum annuum*	1951	+	−	[24]
*P. carotovorum* 98.1	*Solanum tuberosum*	1998	+	+	[27]
*P. carotovorum* RNS 08.42.1A	*Solanum tuberosum*	2008	+	−	V. Hélias' collection
***Dickeya*** ** spp.**					
*D.chrysanthemi* CFBP 2048^T^	*Chrysanthemum morifolium*	1956	+	+	CFBP, type strain
*D. dadantii* 3937	*Saintpaulia ionantha*	1981	+	+	[89]
*D. dianthicola* RNS 04.9	*Solanum tuberosum*	2004	+	+	V. Hélias' collection
*D. solani* RNS 08.23.3.1A	*Solanum tuberosum*	2008	+	+	[27]

* The potential of each strain to induce a tuber soft-rot was evaluated in potato host plant seven days after infection by intra-medulla injection.

# The potential of each strain to induce a hypersensitive response (HR) was evaluated in tobacco non host plant 24 hours after leaves infiltration.

CFBP, Collection Française de Bactéries Phytopathogènes, Institut National de la Recherche Agronomique, Angers, France.

### Growth media and conditions

Liquid cultures were grown in polygalacturonic acid (PGA) mineral salt medium [40] the composition of which has been modified as follows: K_2_HPO_4_, 16.266 g/l; KH_2_PO_4_, 899 mg/l; (NH_4_)_2_SO_4_, 1.2 g/l; MgSO_4_.6H_2_O, 818 mg/l; CaCl_2_, 75 mg/l (pH 8.0) and polygalacturonic acid 4.0 g/l (Potassium salt, Sigma-Aldrich, France). For production of quinolones and auxins, bacteria were also grown in King B and M9 minimal media supplemented with l-tryptophan at 500 µg/ml [36]. Batch cultures were performed under gyratory agitation (180 rpm) at 25°C in Erlenmeyer flasks in which the liquid medium is 10% of the total flask volume. Batch precultures and cultures were made in the same experimental conditions. Bacterial growth was monitored by measuring optical density (OD) at 580 nm. The initial OD_580_ of the cultures was usually 0.05. Each experiment using triplicate independent cultures were repeated at least three times. The filtered supernatants and cells obtained were stored at −20°C. *E. coli* carrying pSB401 strains were grown in LB medium supplemented with tetracycline (10 µg/ml), and maintained on LB agar supplemented with tetracycline.

### Soft-rot test in potato tuber

NaCl-washed *Dickeya* and *Pectobacterium* cell suspensions (cell density ca. 10^8^ cfu/ml in 0.9% NaCl) were prepared from stationary-phase cultures grown in PGA minimal medium. *S. tuberosum* cv. Allians tubers were surface-sterilized and infected by intra-medulla injection (1-cm depth with 10 µl of the above bacterial suspension). The inoculated tubers were incubated in a Minitron incubator (Infors, Massy, France) at 25°C under a relative humidity of 65%±2%. For each strain, five tubers were analyzed and the development of the symptoms was evaluated after 7 days.

### Hypersensitive Response assays in tobacco plants

*Nicotinia tabacum* cv. *Xanthi* XHFD8 was cultivated for 8 weeks under greenhouse conditions (T = 28°C, photoperiod = 16 h). Bacteria were cultivated in PGA minimal medium at 25°C, washed twice with 0.9% NaCl, and resuspended at 2×10^8^ cfu/ml. Tobacco leaves were infiltrated with ca. 100 µl of cell suspensions. The margins of the water-soaked infiltrated areas were marked, and the plants were inspected for HR development at 24 h.

### NAHSL standards, extraction of supernatants and NAHSL assays

NAHSLs were extracted and analyzed as described previously [12], [41]. Briefly, the synthetic standards (Sigma-Aldrich, France) and stock solutions prepared in high performance liquid chromatography (HPLC)-grade ethyl acetate (Fisher Scientific, France) were stored at −20°C. The supernatants (1 ml) were extracted twice with equal volumes of ethyl acetate. The combined extracts were dried over anhydrous magnesium sulfate, evaporated to dryness, dissolved in 500 µl of HPLC-grade ethyl acetate and stored at −20°C until analysis. Components in the ethyl acetate extracts were spotted onto C18 reversed-phase thin-layer chromatography (TLC) Silicagel (Whatman, Maidstone, U.K.) plates and separated with methanol-water (60∶40, v/v), as described by Shaw and associates [42]. NAHSL were detected by overlaying the TLC with the indicator strains essentially as indicated by Cha et al. [43].

Concentrated extracts were analyzed by on-line liquid chromatography mass spectrometry (LC-MS-MS). They were applied to a C18 reverse-phase HPLC column (Agilent Hypersyl ODS, 250×4.6 mm, particle size 5 µm, Interchim, France) using an Agilent Technologies Series 1100 vacuum degasser, LC pump and autosampler (Hewlett Packard, Germany). The elution procedure consisted of an isocratic profile of methanol-water (50∶50, v/v) (Fisher Scientific, France) for 10 min at a flow rate of 0.4 ml/min, followed by a linear gradient from 50% to 90% methanol in water over 15 min, and a isocratic profile over 25 min. The post-column flow was split (1/10) by a micro-splitter valve (Upchurch Scientific, USA) and a mixture of 5 mM ammonium acetate and 0.05% trifluoroacetic acid (Sigma-Aldrich, France) in methanol-water (50∶50, v/v, 150 µl/h) was added using a Cole-Parmer syringe pump. Detection was performed by electrospray ionisation-ion trap mass spectrometry (ESI-MS) using a Bruker Esquire-LC spectrometer (Bruker Daltonic, Germany) under positive-ion conditions. The identification of NAHSLs from supernatant extracts was done by comparison with synthetic standards, based on three criteria: HPLC retention times, MS-MS fragment ions of the molecular [M+H]+ ions (four product ions: the lactone ring *m*/*z* 102, [M+H−101]+ ion corresponding to the acyl chain, [M+H−H_2_O]+ and [M+H−CO]+ ions) and on their relative intensities. Chromatographic peak area of the *m*/*z* 102 ion was measured for quantification [41].

### AI-1 and AI-2 bioluminescence assays

Potato soft-rot strains were grown in PGA minimal medium at 25°C, whereas *E. coli* and *V. harveyi* were cultivated in LB at 37°C and in autoinducer bioassay (AB) medium at 30°C, respectively. Bacterial growth was monitored by measuring OD at 580 nm. After the appropriate incubation period, bacterial cells were removed by centrifugation at 10 000× *g* for 10 min and the resulting supernatant was subsequently filter sterilized through a 0.22 µm pore size filter (Millipore, Billerica, MA) and stored at −20°C. The filtered samples were then analyzed using the biosensor strains *E. coli* DH5α(pSB401) and *V. harveyi* BB170 as previously described [44], [45].

### Detection of the 2-alkyl-4-(1H)-quinolones

Potato soft-rot strains were grown at 25°C in PGA minimal medium or M9 minimal medium supplemented with l-tryptophan at 500 µg/ml. Aliquot of stationary phase cell-free supernatants (10 ml) were twice extracted with 10 ml acidified ethyl acetate, vortexed vigorously and centrifuged 10 000× *g* for 5 min. The organic phase was transferred to a fresh tube and dried to completion under a stream of nitrogen gas. The solute residue was resuspended in 500 µl methanol. Samples of ethyl-acetate extracted culture supernatant were spotted onto a normal phase silica 60_F254_ (Merck) TLC plate that had been previously soaked for 30 min in 5% w/v KH_2_PO_4_ and activated at 90°C for 1 h. HHQ and PQS produced by *P. aeruginosa* H103 strain were used as positive controls. After drying the spots, TLC plates were developed by using dichloromethane∶methanol (95∶5 v/v) as the mobile phase then visualized with a UV transilluminator and photographed.

### Auxins quantification, extraction and characterization

Auxin quantification was performed with a Fe-H_2_SO_4_ reagent [46]. Briefly, bacterial strains were propagated overnight into 5 ml of King B medium and transferred in PGA minimal medium or M9 minimal medium supplemented with l-tryptophan at 500 µg/ml. Samples were taken at different points of the growth curves, 1 ml aliquot of culture supernatant was mixed vigorously with 4 ml of Salkowski's reagent (150 ml of concentrated H_2_SO_4_, 250 ml of distilled H_2_O, 7.5 ml of 0.5 M FeCL_3_·6H_2_O), and the absorbance at 535 nm was measured. Auxin production was measured as IAA equivalents using a standard curve of IAA.

Aliquots of sterilized supernatants (8.5 ml) were acidified to reach a pH 3 using HCl (37%). Supernatants were extracted with 4 ml acidified ethyl acetate, vortexed vigorously and incubated overnight at 4°C. Supernatants were extracted twice with 3 ml acidified ethyl acetate during 2 hours at 4°C. The organic phase was transferred to a fresh tube after centrifugation at 10 000× *g* for 5 min, then was dried to completion under a stream of nitrogen gas. The solute residue was resuspended in 1 ml methanol/water (v/v, 80/20). Concentrated extracts were analyzed by HPLC. They were applied to a C18 reverse-phase HPLC column (C18 Atlantis, 150×4.6 mm, particle size 3 µm, Waters, France) using an Agilent Technologies Series 1100 vacuum degasser, LC pump and autosampler (Hewlett Packard, Germany). Mobile phase contained purified water 18 MΩ/H_3_PO_4_ (1/1000) (A) and acetonitrile (B). The gradient elution was as follows: 5–90% B at 0–25 min at a flow rate of 0.5 ml/min; 90% B at 33–37 min at a flow rate of 1 ml/min; 90–5% B at 33–37 min at a flow rate of 0.5 ml/min; re-equilibrium was 20 min; the total run time was 57 min. Detection was performed by Chromeleon. The identification of the putative auxins in supernatant extracts was done by comparison with synthetic standards, based on HPLC retention times at 325 nm. For quantification, chromatographic peak area was measured and compared to standards. Seven indolic compounds were studied: IAA, indole-3-pyruvic acid (IPyA), indole-3-acetamide (IAM), indole-3-butyric acid (IBA), indole-3-propionic acid (IPA), kynurenic acid (KA) and l-tryptophan. All this compounds were purchased from Sigma-Aldrich (France).

### GABA detection by immunoassay

Soft-rot strains were grown in PGA minimal medium at 25°C. After the appropriate incubation period, bacterial cells were removed by centrifugation at 10 000× *g* for 10 min and the resulting supernatant was subsequently filter sterilized through a 0.22 µm pore size filter (Millipore, Billerica, MA) and stored at −20°C. The filtered samples were analyzed by GABA Elisa test in accordance with instructions from GABA Elisa kit (Labor Diagnostika Nord & Co., KG).

### RNA extraction and reverse transcription-PCR (RT-PCR)

Total RNA was isolated from ∼2×10^9^ cells of bacterial cultures using hot acidic phenol. A lysis buffer (0.02 M sodium acetate, pH 5.5, 0.5% (w/v) SDS, 1 mM EDTA) allowed the obtaining of cell lysates subjected to three consecutive phenol extractions, followed by a chloroform extraction. Total RNAs were precipitated with 100% ethanol (2∶1, v/v) and 1 M sodium acetate (1∶10, v/v), and resuspended in RNase-free water. DNase I treatment using RNAse-free DNase I (Ozyme, Saint-Quentin en Yvelines, France) allowed removal of contaminating DNA. Quality and concentration of RNA samples were checked by agarose gel electrophoresis, and using a Nanodrop spectrophotometer (Bio-Rad Laboratories). Absence of genomic DNA contamination was confirmed by PCR. RT-PCR was performed with 5 ng RNA as template using Transcriptor one step RT-PCR kit (Roche, Meylan, France) according to the manufacturer's recommendations. Because of the high abundance of 16S rRNA, RNA samples were diluted 100 fold more when analyzing 16S rRNA reference gene expression. After optimization of the RT-PCR protocol according to the abundance of the respective mRNAs, 35 and 40 cycles were used for all genes of *Pectobacterium* and *Dickeya* strains, respectively.

The set of primers used for amplification of mRNA encoding the synthases from S-adenosylmethionine (SAM), AI-1, AI-2 and IAA precursor are listed in Supporting Information (Table S1). The corresponding genes are respectively named *metK*, *luxI*, *luxS* and *iaaM*. The primer sequences were deduced from the *P. atrosepticum* SCRI1043, *P. carotovorum* PC1, *D. dadantii* 3937 and *D. zeae* Ech586 genomes (GenBank accession number NC_004547, NC_012917, NC_014500, and NC_013592, respectively). For calibration, 16S rRNA and *recA* were used as references [47],[48]. The corresponding primers were designed from a multiple sequence alignment and allowed to amplify a unique 591-bp and 974-bp fragment, respectively. All amplified fragments displayed the expected size.

## Results

### Characterization of signal molecules produced by potato soft-rot bacteria

In this study, the potato was chosen as the model host-plant because of its sensitivity to a broad range of soft-rot bacteria and its key role in the world global food system as it is the world's fourth most produced food commodity [23]. Moreover, the large-scale cultivation of this plant facilitates the recurrent isolation of many isolates including isolates used in this study. A panel of twelve bacteria was compared for signal production (Tables 2 and 3). It was composed of three type strains which are very ancient isolates used as international taxonomic references of *P. atrosepticum*, *P. carotovorum* and *Dickeya chrysanthemi* (formerly *Erwinia chrysanthemi*) species [49]. Three reference strains which are commonly used for virulence studies were also included in this panel: (*i*) the psychrotroph strain *P. atrosepticum* CFBP 6276 known both for its virulence on the potato and for the unusual ability among this species to induce HR in non host plant [12], [40], (*ii*) the strain *P. carotovorum* EC153, isolated from rotted bell pepper fruit shipped from Mexico and which NAHSL production and virulence were exceptionally enabled at elevated temperatures above 34°C [24], and (*iii*) *D. dadantii* 3937, a mesophile strain isolated from the African violet, widely used as a model system for research on the molecular biology and pathogenicity of soft-rot bacteria and which genome has recently been published [50]. Finally, six recent isolates from potato blackleg wounds were added to this sampling. They represent the different soft-rot potato species currently encountered in European soils, including an emerging *Dickeya* clade, provisionally called *D. solani* [26], [27], [31]. All *P. carotovorum* strains of this collection belong to *carotovorum* subspecies division.

**Table 3:** Characterization of signaling molecules produced by potato soft-rot pathogens.

Strains	AI-1 (NAHSL)*(ng/OD_580_)					AI-2#	Quinolones°		Auxins† (ng/OD_580_)				GABA§
	3-oxo-C6-HSL	3-oxo-C8-HSL	3-oxo-C10-HSL	C6-HSL	C8-HSL	Activity	HHQ	PQS	IAA	IPA	IBA	KA	
***Pectobacterium atrosepticum***													
*P. atrosepticum* CFBP 1526^T^	59±5	1475±125	≤5±2	≤5±2	67±10	**+**	**−**	**−**	**−**	**−**	**−**	**−**	**−**
*P. atrosepticum* CFBP 6276	92±10	2300±250	10±3	≤5±2	100±14	**+**	**−**	**−**	**−**	**−**	**−**	**−**	**−**
*P. atrosepticum* 100T	26±6	650±100	**−**	≤5±2	34±4	**+**	**−**	**−**	**−**	**−**	**−**	**−**	**−**
*P. atrosepticum* RNS 08.30.1A	24±4	600±150	**−**	≤5±2	41±6	**+**	**−**	**−**	**−**	**−**	**−**	**−**	**−**
***Pectobacterium carotovorum***													
*P. carotovorum* CFBP 2046^T^	2500±125	100±5	**−**	≤5±2	**−**	**+**	**−**	**−**	**−**	**−**	**−**	**−**	**−**
*P. carotovorum* EC153	25±4	625±100	**−**	36±3	**−**	**+**	**−**	**−**	**−**	**−**	**−**	**−**	**−**
*P. carotovorum* 98.1	2225±200	89±8	**−**	≤5±2	**−**	**+**	**−**	**−**	**−**	**−**	**−**	**−**	**−**
*P. carotovorum* RNS 08.42.1A	950±225	38±9	**−**	≤5±2	**−**	**+**	**−**	**−**	**−**	**−**	**−**	**−**	**−**
***Dickeya*** ** spp.**													
*D. chrysanthemi*CFBP 2048^T^	950±75	23±3	**−**	≤5±2	**−**	**+**	**−**	**−**	11 990±1000	**−**	106±33	78±5	**−**
*D. dadantii*3937	300±25	12±1	**−**	≤5±2	**−**	**+**	**−**	**−**	12 910±1440	364±71	78±7	54±20	**−**
*D. dianthicola*RNS 04.9	125±25	5±1	**−**	≤5±2	**−**	**+**	**−**	**−**	35 950±2350	22±10	90±30	67±20	**−**
*D. solani*RNS 08.23.3.1A	50±5	≤5±2	**−**	≤5±2	**−**	**+**	**−**	**−**	876±190	**−**	82±2	66±10	**−**

* Production of different N-acyl homoserine lactones (NAHSL) was determined by thin-layer chromatography (TLC) and HPLC-MS/MS. For quantification, NAHSL were extracted from PGA minimal medium culture at late exponential phase (optimal production).

# Autoinducer-2 (AI-2) activity was determined using biosensor *V. harveyi* BB170.

° Production of 4-hydroxy-2-heptylquinoline (HHQ) and Pseudomonas quinolone signal (PQS) were determined by TLC.

† Production of indole-3-acetic acid (IAA), indole-3-propionic acid (IPA), indole-3-butyric acid (IBA) and kynurenic acid (KA) were determined by HPLC-UV. For quantification, indolic compounds were extracted from M9 minimal medium supplemented with l-tryptophan culture at stationary growth phase (optimal production).

§ Production of γ-amino butyric acid (GABA) was determined by ELISA test.(+) positive detection by the biosensor; (−) not detected or below threshold.

All strains were able to induce tissue maceration seven days after potato tuber inoculation. In addition, all the *Dickeya* strains were able to induce an HR in tobacco, but only half of *Pectobacterium* strains had this capacity (Table 2). This result is consistent with recent work published by Kim et al. [51] reporting that deletions can occur in the locus encoding the type III secretion system in *Pectobacterium* strains. However, no difference on potato virulence was observed between HR-negative and HR-positive *Pectobacterium* strains (Table 2).

Characterization of quorum sensing signal molecules was done on bacterial cells cultivated at 25°C. Two minimal media were used for that purpose. The mineral salt medium supplemented with PGA was used in each assay. This vegetable compound induces the synthesis of virulence factors involved in plant disease or resistance, which were shown to be under AI-1 QS control [12]. The M9 medium was supplemented with l-tryptophan, a precursor of numerous auxins and quinolones [52], [53]. The tryptophan was used as the sole source of carbon for bacterial growth during auxin and 2-alkyl-4-(1*H*)-quinolone detection assays. Produced NAHSLs were identified and quantified by TLC and HPLC coupled with mass spectrometry. In these conditions, all *P. atrosepticum* strains and the *P. carotovorum* EC153 strain produced mainly *N*-3-oxo-octanoyl-l-HSL (3-oxo-C8-HSL) and minor quantities of *N*-octanoyl-l-HSL (C8-HSL) and *N*-3-oxo-hexanoyl-l-HSL (3-oxo-C6-HSL) (Table 3). They also synthesized traces of *N*-hexanoyl-l-HSL (C6-HSL) and *N*-3-oxo-decanoyl-l-HSL (3-oxo-C10-HSL). The *P. atrosepticum* CFBP 6276 and 1526^T^ strains were the largest producers of 3-oxo-C8-HSL with amounts superior to 1 µg/OD_580_. The other *P. atrosepticum* strains and *P. carotovorum* EC153 displayed lower production of NAHSL with about 600 ng/OD_580_ of 3-oxo-C8 plus amounts generally twenty-fold lower of C8-HSL. The three other *P. carotovorum* strains and all *Dickeya* spp. strains produced mainly 3-oxo-C6-HSL. Comparing these two bacterial genera revealed that the NAHSL production is lower in *Dickeya* than in *Pectobacterium* (Table 3).

The production of AI-2 was indirectly assessed by detecting AI-2 signal activity. Supernatants from all soft-rot pathogens studied here were tested for their ability to induce luminescence in *V. harveyi* BB170. In each case, the addition of 10% cell-free culture induced a luminescence emission within the reporter strain. As the positive control *V. harveyi* BB120, all strains are able to produce extracellular AI-2 activity (Table 3). In contrast, the negative control (*E. coli* DH5α) did not induce luminescence of the reporter strain.

Salkowski's reaction showed that only *Dickeya*. spp. can produce indole compounds. This production only occurred when bacteria were grown in the minimal medium supplemented with l-tryptophan. The characterization of the different auxins was carried out by HPLC-UV and compared to standards (Table 3). IAA was always the major auxin extracted from culture supernatants. Among all tested supernatants, the highest IAA amount was observed in the supernatant of isolate *D. dianthicola* RNS 04.9, while the lowest was found in the supernatant of isolate *D. solani* RNS 08.23.3.1A. All the *Dickeya* strains synthesized IBA and KA in amounts 10- to 700-fold lower than the IAA ones. Finally, traces of IPA are also detected in *D. dadantii* and *D. dianthicola* strains (Table 3).

The ability of soft-rot pathogens to produce 2-alkyl-4-(1*H*)-quinolones and GABA signaling molecules was also assessed using TLC visualized under UV and ELISA, respectively. We failed to detect HHQ, PQS or GABA in culture supernatants of all soft-rot pathogens, indicating that either these molecules were not produced or that they were produced at amounts lower than the detection limits estimated to be about 20 nmol for quinolones and 75 ng for GABA in our conditions (data not shown).

### Kinetics of AI-1, AI-2 and IAA signal molecules production

**Figure 1:**
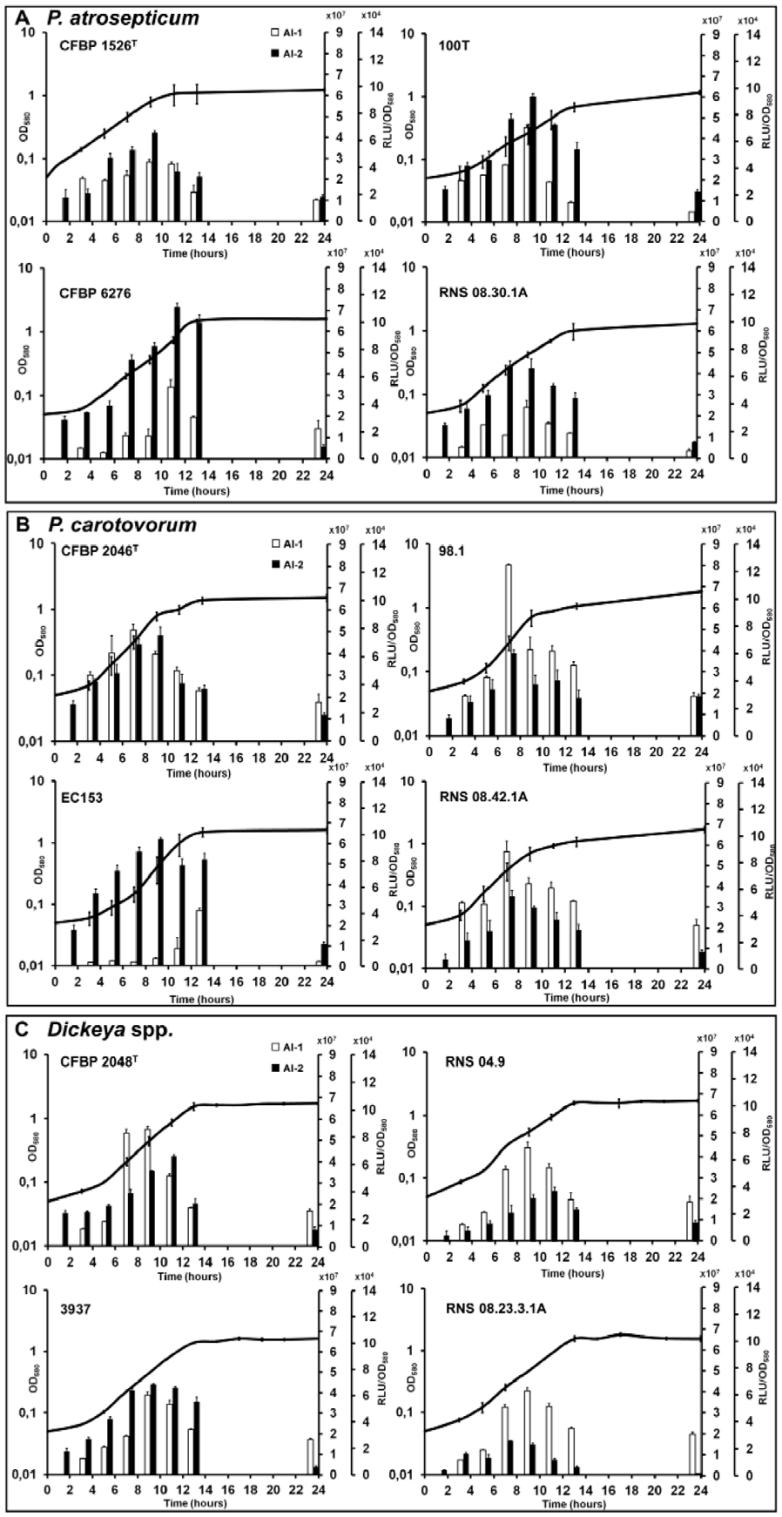
Kinetics of AI-1 and AI-2 activities measured in the supernatant of potato soft-rot. *Pectobacterium atrosepticum* (A), *Pectobacterium carotovorum* (B) and *Dickeya* spp. (C) using a bioluminescence assay with *Escherichia coli* DH5α(pSB401) and *Vibrio harveyi* BB170 reporter strains, respectively. Bacterial growth was monitored by measuring optical density (OD) at 580 nm. For each point, at least 3 independent cultures in PGA minimal medium were analyzed, with standard deviation shown. Legend: white bars, AI-1 activity measurable with the first right Y-axis; black bars, AI-2 activity measurable with the second right Y-axis; RLU, relative luminescence unit.

Free-cell supernatants were prepared from cultures at different ODs in PGA minimal medium. The kinetics of AI-1 and AI-2 production were then determined by bioluminescence assay using as reporters *E. coli* DH5α(pSB401) and *V. harveyi* BB170 strains, respectively. Results show a concomitant AI-1 and AI-2 production by all *P. atrosepticum* strains (Fig. 1A). AI-1 and AI-2 activities increased from the second half of the logarithmic phase with a peak between 8 and 12 hours, and then declined during the stationary phase, which started between 10 and 14 h in this culture medium (Fig. 1A). This shows the transient activities of AI-1 and AI-2 in all soft-rot bacteria whatever their origin. The two *P. carotovorum* isolates 98.1 and RNS 08.42.1A (Fig. 1B), the *D. dadantii* 3937 strain and the *D. solani* RNS 08.23.3.1A isolate (Fig. 1C) displayed production profiles similar to the ones of *P. atrosepticum* (Fig. 1A). On the other hand, *P. carotovorum* CFBP 2046^T^ and EC153 presented atypical patterns with an early or a delayed AI-1 activity, respectively (Fig 1B). For *Dickeya* spp. strains, while AI-1 activity was always optimal at 9 h, AI-2 activity was more or less offset in time relative to that of AI-1 (Fig. 1C).

**Figure 2:**
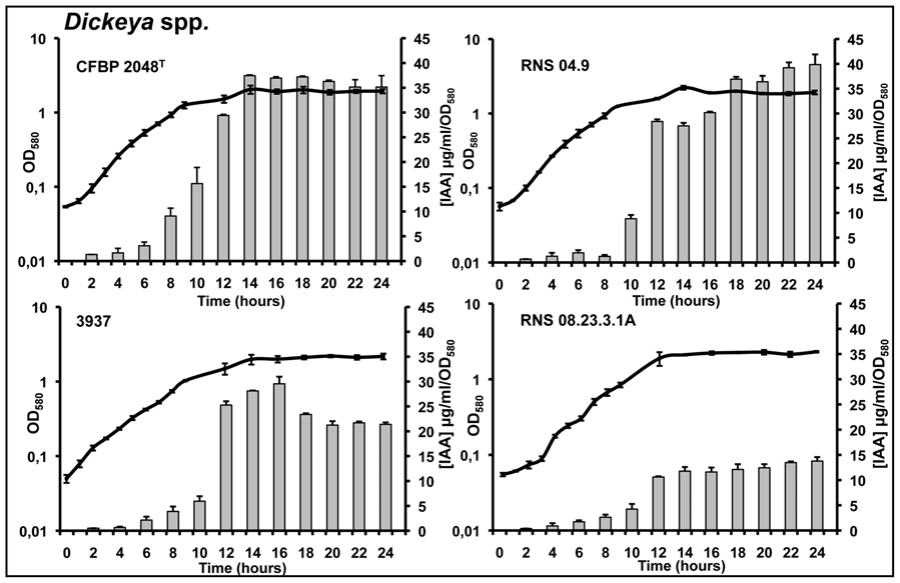
Kinetics of IAA-like production measured in the supernatant of potato soft-rot *Dickeya* spp. Indolic compounds were quantified by a colorimetric method with Salkowski's reagent. Bacterial growth was monitored by measuring optical density (OD) at 580 nm. For each point, at least 3 independent cultures in M9 minimal medium supplemented with l-tryptophan (500 µg/ml) were analyzed, with standard deviation shown.

The production of auxins only occurred in *Dickeya* spp. strains grown in minimal medium supplemented with l-tryptophan. It was studied by a rapid colorimetric method using Salkowski's reagent. Among indolic compounds detected by this method, IAA is one of the most reactive [46]. According to our HPLC-UV results, the recorded concentrations of indolic compounds reflect essentially IAA concentrations found in culture supernatants. IAA-like concentrations increased from the middle of the exponential phase to reach their maximum values in the stationary phase (Fig. 2). Unlike AI-1 and AI-2 molecules, indole compounds persist at high concentrations throughout the stationary phase (Fig. 2), suggesting a considerable stability of the IAA-like molecules and/or the absence of a degradation mechanism.

### Expression of AI-1, AI-2 and IAA synthase genes in soft-rot bacteria

**Figure 3:**
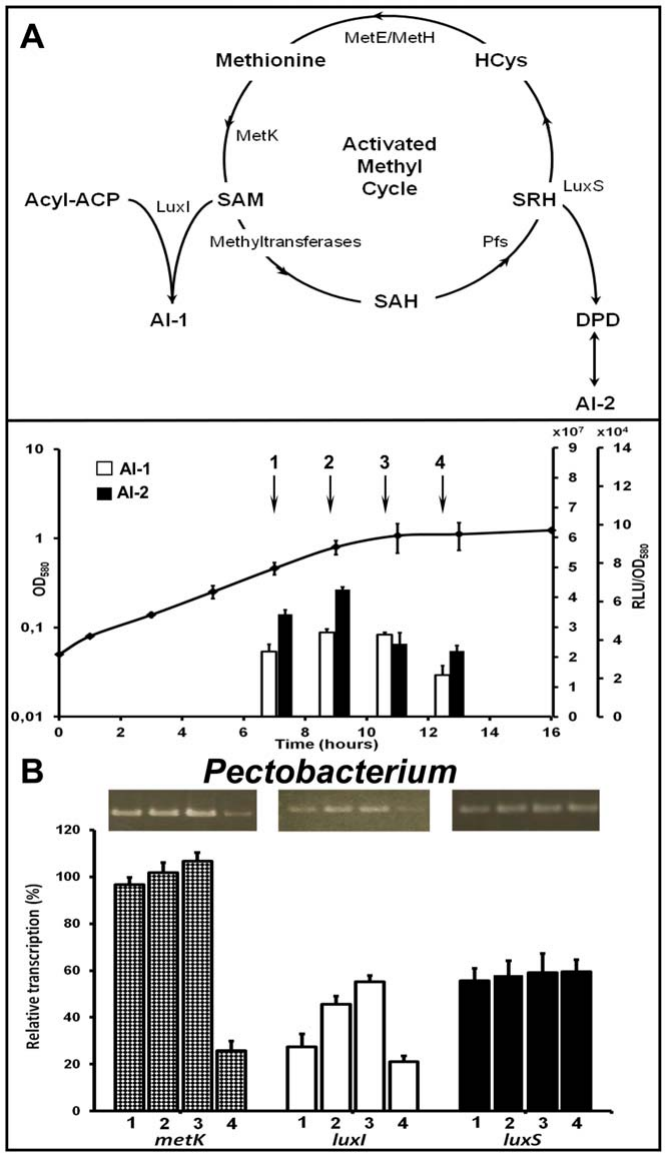
Kinetics of *metK*, *luxI* and *luxS* gene expression involved in AI-1 and AI-2 production by *P. atrosepticum* strain CFBP 1526^T^. (**A**) AI-1 and AI-2 biosynthetic pathways are schematized from references [2], [67], [81]. The synthesis of S-adenosylmethionine (SAM), a key molecule precursor of both AI-1 and AI-2 signals, is catalyzed by the SAM synthase (MetK), which activates the methyl group of methionine after ATP hydrolysis. AI-1 signal is generated by LuxI enzyme from SAM and various acyls-ACP that determine the structural traits of each *N*-acyl homoserine lactone (NAHSL). AI-2 signal is generated as a by-product of the activated methyl cycle: S-adenosylhomocysteine (SAH) is produced by the action of methyltransferases, and is then converted to S-ribosylhomocysteine (SRH) by the nucleosidase Pfs. The enzyme LuxS converts SRH to homocysteine (HCys) and 4,5-dihydroxy 2,3-pentanedione (DPD), which spontaneously cyclizes into several furanones, including AI-2. Homocysteine is then recycled back to methionine using methionine synthases (MetH or MetE). (**B**) Abundances of *metK* (checkered bars), *luxI* (white bars) and *luxS* (black bars) mRNAs were determined by RT-PCR experiments on RNA extracts from cells grown in PGA minimal medium culture and harvested at mid-exponential phase (**1**), late exponential phase (**2**), during the transition from exponential to stationary phases (**3**) or early stationary growth phase (**4**) followed by electrophoresis on 1% (m/v) agarose gels. Results were expressed as a ratio: synthase transcripts vs. 16S transcripts. The corresponding kinetics of AI-1 and AI-2 activities in the supernatant of potato soft-rot *Pectobacterium* strain are shown at the top.

To determine if the variations of signal production according to growth phase could result from expression modifications of the corresponding synthase genes, levels of specific transcripts have been estimated by semi-quantitative RT-PCR. Among them, *luxI* and *luxS* genes were chosen because they encode the enzyme catalyzing the last step in the pathway for AI-1 and AI-2 biosynthesis, respectively. The gene *metK* was added to this study because its product is responsible for the intracellular accumulation of SAM, a precursor common to AI-1 and AI-2 anabolic pathways (Fig. 3A). In the IAA synthesis pathway, only present in *Dickeya* strains (Fig. 2), tryptophan is first converted to indole-3-acetamine (IAM) by the IaaM tryptophan-2-monooxygenase, and IAM is then converted to IAA by the IaaH hydrolase [36], [54]. *iaaM* expression was followed during the bacterial growth, because it has been previously demonstrated that a *iaaM* gene disruption, but not a *iaaH* gene mutation, reduces the production of pectinolytic enzymes and effectors in strain *D. dadantii* 3937 [36].

**Figure 4:**
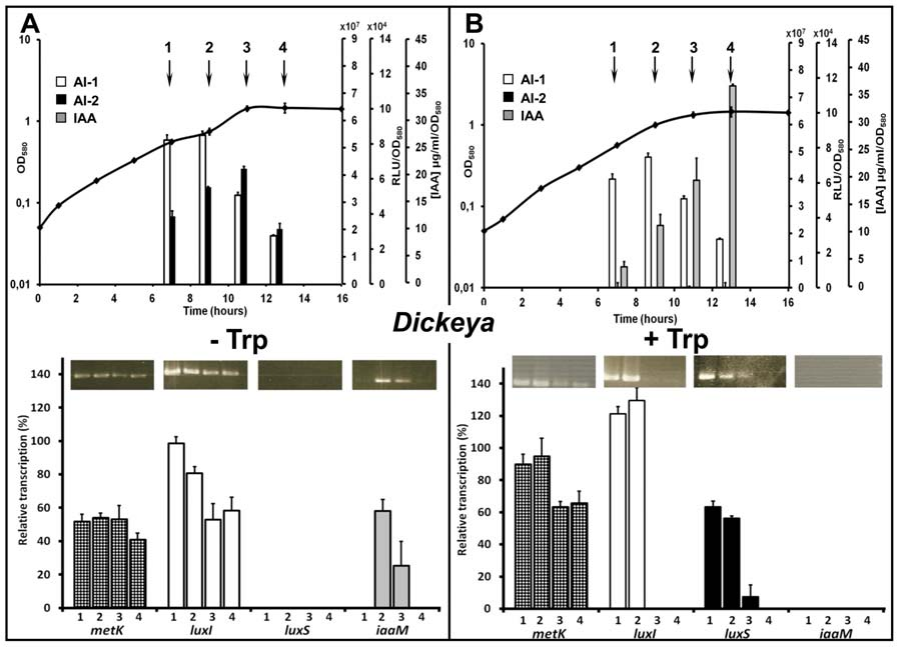
Kinetics of *metK*, *luxI*, *luxS* and *iaaM* gene expression involved in AI-1, AI-2 and IAA production by *D. chrysanthemi* strain CFBP 2048^T^. AI-1 and AI-2 biosynthetic pathways are described in the legend to Fig. 3. A third signal, the indole-3-acetic acid (IAA) is synthesized by the indole-3-acetamine (IAM) pathway, in which l-tryptophan is first converted to IAM by the key enzyme of this pathway, the IaaM tryptophan-2-monooxygenase. IAM is then converted to IAA by IaaM hydrolase [36], [54]. Abundances of *metK* (checkered bars), *luxI* (white bars), *luxS* (black bars) and *iaaM* (grey bars) mRNAs were determined by RT-PCR experiments on RNA extracts from cells grown in PGA minimal medium (**A**) or M9 minimal medium supplemented with l-tryptophan (500 µg/ml) (**B**) and harvested at mid-exponential phase (**1**), late exponential phase (**2**), during the transition from exponential to stationary phases (**3**) or early stationary growth phase (**4**) followed by electrophoresis on 1% (m/v) agarose gels. Results were expressed as a ratio: synthase transcripts vs. 16S transcripts. The corresponding kinetics of AI-1 and AI-2 activities and IAA production in the supernatant of potato soft-rot *Dickeya* strain are shown at the top.

The type strains were chosen as models for this study. As the two *Pectobacterium* type strains yielded similar results, only *P. atrosepticum* CFBP 1526^T^ and *D. chrysanthemi* CFBP 2048^T^ data are presented here. RNAs were extracted from cells under the same culture conditions as those used for signal molecule extractions, *i.e.* in PGA or l-tryptophan supplemented media and at four different points during the bacterial growth (mid-exponential phase, late exponential phase, transition to stationary phase and early stationary phase). In these conditions, the expression of the two reference genes (16S rRNA and *recA*) was stable under all culture conditions whatever the growth phase. The variation in *metK* and *luxI* mRNA abundances is consistent with that of AI-1 activity levels throughout the bacterial growths. For both strains and in each culture condition, *metK* and *luxI* transcript amounts are generally more abundant at the end of the exponential phase of growth (Fig. 3B and 4). In contrast, the *luxS* expression varied depending on the bacterial genus. In *Pectobacterium*, *luxS* was expressed constitutively throughout the bacterial growth (Fig. 3B). Because they vary simultaneously in the same way, the kinetic of the AI-2 activity seems to be under the influence of *metK* transcription responsible for the SAM synthesis that occurs upstream of the metabolic pathway of AI-2 production. In *Dickeya* grown in PGA minimal medium, *luxS* expression is transient and at its most intense in the middle of exponential phase (Fig. 4A). Our results showed a variation of signals and synthases generation according to the carbon source available for the bacteria. In the absence of tryptophan (Fig. 4A), *iaaM* mRNA was not detected, explaining the lack of IAA production, whereas AI-1 and AI-2 activities showed the same transient profile as that of *metK*, *luxI* and *luxS* transcripts. In the presence of tryptophan (Fig. 4B), *iaaM* expression and IAA synthesis rose while the *luxS* expression and the AI-2 production were inhibited. If the production of *iaaM* transcript amount is transient, that of IAA continues to increase in culture until reaching a plateau, showing that this substance is accumulated in the extracellular medium (Fig. 2 and 4B).

## Discussion

Communication systems play an important role throughout bacterial interactions with the host (virulence, symbiosis) and the environment (biofilm development). Fundamental knowledge on these communication systems are therefore of particular interest to the community of microbiologists and could find application in improving human health, food safety and crop yields [2]–[5]. As an example, novel biocontrol strategies based on the disruption of bacterial communication are being developed [23]. One of them consists in characterizing the signaling molecules used by the target pathogen, and then using harmless structural analogs to stimulate microflora able to degrade both molecule families [18]. Because the steps between the transfer of laboratory knowledge to the registration of biocontrol formulations are time- and money-consuming, the identification of the signals involved in host virulence and the study of their representation in the current and emerging pathogens are prerequisite for a global success of this approach. Unfortunately, there are random limited numbers of *Pectobacterium* and *Dickeya* genomes available: they do not reflect the diversity of the current fields isolates nor correspond to emergent strains. The signaling molecule production was therefore checked in a significant number of strains rather than relying on an *in silico* study only.

NAHSL-based QS is the best known autoinducer system, including in soft-rot bacteria [15]. The production of signal molecules involved in this QS system has indeed been observed very early in *P. carotovorum* [14], [16] and then in *D. dadantii* [22]. More recently, Chatterjee et al. [55] defined two classes of QS systems, using mainly 3-oxo-C8-HSL (Class I) or 3-oxo-C6-HSL (Class II) as autoinducer. This duality is linked to the structural characteristics of the NAHSL synthase (LuxI-type protein) encountered in each *Pectobacterium* strain [56], [57]. It is also related to the existence in *Pectobacterium* of two types of NAHSL receptors (LuxR-type proteins) that have a different affinity for each of the two NAHSLs [58]–[60]. Our results show that NAHSLs production is widespread within both old and emerging soft-rot bacteria isolates. Minor species of NAHSLs, likely to be less specific products of LuxI synthase or catabolites resulting from NAHSLs turnover, coexist with 3-oxo-C6-HSL and 3-oxo-C8-HSL, the two molecules identified as true QS signals [13], [56], [57]. However, the nature of these signals seems to be dependent on the bacterial genus or species. *P. atrosepticum* strains produce 3-oxo-C8-HSL while *Dickeya* spp. produce 3-oxo-C6-HSL as signal molecules. These findings are consistent with previous studies made on other soft-rot strains [21], [43], [55], [61]. There is however an exception to this rule: to our knowledge, *P. atrosepticum* SCRI1043 is the only studied strain harboring the QS signaling system of class II and unfortunately it is today the only strain with a sequenced genome [62], [63]. The class I and II distinction is not easy for *P. carotovorum* strains. Although a majority of strains produce 3-oxo-C6-HSL, at least *P. carotovorum* EC153 and SCC3193 strains produce 3-oxo-C8-HSL as signaling molecules [24], [56], [64]. These differences could be explained by the strong heterogeneity of the *P. carotovorum* species and the diversity of their hosts and environmental niches [65], [66].

AI-2 is another type of autoinducer described as a mediator of interspecies communication in Gram-positive and Gram-negative bacteria [5], [67]. Today, the fine structure of AI-2 has been only resolved in *V. harveyi* (as furanosyl borate diester) and *Salmonella enterica* ser. typhimurium (as 2R,4S tetrahydroxy-tetrahydrofurane) after co-crystallization of AI-2 with their binding protein [68], [69]. Nevertheless, the ‘AI-2’ term refers more generally to furanones derived from the spontaneous cyclization of 4,5-dihydroxy 2,3-pentanedione (DPD) with or without boron, and which induce bioluminescence in the *V. harveyi* bioassay [67]. The production of AI-2 and the presence of the AI-2 synthase (LuxS-type protein) have been demonstrated in three *Pectobacterium* strains in 2006. In two of them, ATCC39048 and SCC3193, a *luxS* mutation affects bacterial motility, virulence and the progression of disease symptoms during early stages of infection through the modulation of the expression of pectinolytic enzymes [33], [34]. In contrast, virulence does not seem to be affected in a *luxS* derivative of *P. atrosepticum* SCRI1043 [33]. For the first time, we are reporting AI-2 synthesis within four *Dickeya* species and, more consequently, the ubiquitous production of this signaling molecule by potato soft-rot pathogens. Provided that physiological function is confirmed, this autoinducer therefore can be a novel target for overall pathogen control in plants.

The 2-alkyl-4-(1*H*)-quinolones form a third family of QS signaling molecules recently reviewed by Heeb et al. [70], in which PQS and HHQ are the best known members. These autoinducers were involved in regulation and expression of multiple virulence genes in *P. aeruginosa* and related *γ-Proteobacteria* [71]–[73]. In addition, the production of quinolones and NAHSL are closely linked to each other within *P. aeruginosa*. In this species, the effectiveness of the two QS systems depends on synchronous interactions of these molecules [74]. As soft-rot bacteria are also *γ-Proteobacteria* which synthesize NAHSL, we attempted to detect, in vain, the production of HHQ and PQS with or without l-tryptophan precursor in the culture medium. It is unlikely that these signals are used by the six bacterial species studied. Consistently, the available *Pectobacterium* and *Dickeya* genomes are devoid of genes for enzymes similar to known HHQ and PQS synthases (data not shown).

Auxins are well known phytohormones, among which the most famous member is IAA [54]. IAA is also produced by bacteria and its synthesis can be autoinduced [38], [52]. Several recent reports indicate that IAA and some other auxins can function as signal molecules with a direct effect on bacterial physiology for example in the transition from a low- to a high-cell-density state [39], [53], [54]. Indole compounds have therefore the potential to be QS molecules although their perception and transport mechanisms have not yet been elucidated in bacteria [39], [54]. IAA synthesis has been shown to be required for full virulence of *D. dadantii* 3937 in Saintpaulia host plant, influencing the local maceration symptoms in leaves but not the bacterial growth in plants [36]. As IAA controls virulence gene expression via the RsmA/*rsmB* pathway in a similar manner to NAHSL in *Pectobacterium*, IAA has been suggested to play an analogous role in *D. dadantii* [75]. Our results show that all tested *Dickeya* species produce IAA as the major auxin. On the contrary, in our conditions, the fact that none of the *Pectobacterium* strains is able to synthesize a detectable indolic compound means an important distinction between the two bacterial genera responsible for potato soft-rot. To date, six IAA anabolic pathways were identified in bacteria [54]. Among them, only the IAM pathway was identified in *D. dadantii* 3937 [36]. In this pathway, tryptophan is first converted by *iaaM* gene product, which plays a key role. The *iaaM* expression is transient, whereas IAA accumulates in the culture supernatant during the stationary phase. Furthermore, *iaaM* transcription and IAA production occur only in the presence of l-tryptophan. This suggests that tryptophan can act both as a precursor and as an inducer of durable signal molecule synthesis.

GABA is also an extracellular signal molecule involved in multiple plant-microorganism interactions [76]. GABA is rapidly accumulated by plants in response to a variety of stresses including bacterial or fungal infection at wounding sites [77]. In numerous *γ-Proteobacteria*, GABA is synthesized by glutamate decarboxylase that catalyses the irreversible α-decarboxylation of glutamate [78]. This molecule can affect the behavior and the QS system of the pathogen. For example, GABA and IAA signaling pathways are suspected to interfere with the virulence program of the plant pathogen *Agrobacterium tumefaciens* [37]. GABA also may modulate bacterial QS, thereby affecting the virulence of *Agrobacterium* by controlling the level of intracellular NAHSL [35], [79]. In our test conditions, *Pectobacterium* and *Dickeya* spp. do not produce detectable amounts of GABA. Moreover, the absence of glutamate decarboxylase-like enzymes among the proteins encoded by the known genomes of *Pectobacterium* and *Dickeya* strengthens the notion that this amino acid does not play a role in the soft-rot bacteria communication (data not shown).

To our knowledge, the interactions between signal production pathways of soft-rot bacteria were not previously studied. We investigated the expression of key genes involved in metabolic pathways of AI-1, AI-2 and IAA, including the expression of SAM synthase, because AI-1 and AI-2 productions are dependent on the available SAM pool. In the cell, the primary roles of SAM are to serve as a precursor for the membrane phospholipid phosphatidyl-choline and as methyl group donor for methylation of nucleic acids and other molecules. In addition, SAM is a substrate for the synthetic reactions leading to AI-1 and AI-2 molecules [80]. In the bacterial cytosol, the SAM synthase (MetK) is responsible for intracellular generation of SAM, while the NAHSL synthase (LuxI) is directly responsible for the net consumption of a part of the SAM pool. Another part of this pool enters in the activated methyl cycle in which the AI-2 synthase (LuxS) yields the 4,5-dihydroxy 2,3-pentanedione (DPD), which then spontaneously cyclizes to yield AI-2. Here, there is no SAM consumption since the second product of this reaction, the homocysteine, is recycled to favor SAM regeneration [67], [81], [82]. Our results show that only 7 of 12 soft-rot strains have concomitant kinetics of AI-1 and AI-2 activity. Therefore there is no obligate link between the production kinetics of these two signals throughout the bacterial growth. These observations are confirmed by differences in AI-1 and AI-2 synthase gene transcription in *Pectobacterium*. If the NAHSL production appears to be directly linked to the NAHSL synthase expression, the AI-2 one seems rather under the control of the activated methyl cycle and the SAM synthase expression. Nevertheless, we confirm the hypothesis that one of the mechanisms involved in the regulation of *Pectobacterium* AI-1 QS consists of controlling NAHSL synthase production by modulating the amounts of *luxI* transcripts [13]. Another outcome of the present work is that AI-2 and IAA signaling pathways do not seem to act simultaneously. The switch occurs in *Dickeya* bacteria according to tryptophan amino acid availability in the microenvironment. Under conditions with sufficient amount of tryptophan, *luxS* mRNA is undetectable (whereas the enzyme MetK remains active to provide the necessary SAM) while *iaaM* expression and IAA production are induced. At low tryptophan concentration, only AI-2 production takes place. Tryptophan is exuded from plants in large quantities within the rhizosphere [83]. It is also involved in IAA synthesis that occurs in plant cells during lateral root initiation. Therefore it is widely released by older root sections resulting from lateral root perforation of the root epidermis [84]. The release of significant amounts of indole compounds (*i.e.* plant tryptophan or IAA) may therefore induce a change in the type of QS-based communication, thereby revealing to *Dickeya* the proximity of its privileged infection areas, such as root hair emergence or wounded roots. This could partly explain why *Dickeya* isolates appear to be better root invaders than *Pectobacterium* isolates (V. Hélias, *unpublished data*).

In conclusion, we showed that potato soft-rot bacteria possess the common ability to produce a substituted NAHSL and a DPD-derived furanone as signal molecules with a transient activity. In addition, *Dickeya* spp. can produce stable quantities of IAA instead of the furanone autoinducer in tryptophan rich condition. All these signaling molecules have been identified for the first time in an isolate belonging to the novel *D. solani* species. To this step of our knowledge, results designate 3-oxo-C6/8-HSL as the main targets for a global biocontrol of soft-rot bacteria communications, including those of emerging isolates. Even if the production of signaling molecules was shown in media that mimic plant wound conditions and promote the synthesis of induced virulence factors, the next stage of this work will be to assess the role of each bacterial signaling pathway *in planta* virulence.

## Supporting Information

Table S1 **Primers used for RT-PCR of potato soft-rot pathogens.**
(DOC)
