# Supplementary material for: Quorum Sensing Signaling Molecules Produced by Reference and Emerging Soft-Rot Bacteria (Dickeya and Pectobacterium spp.)
Source: PLoS One. 2012 Apr 23;7(4):e35176. doi: 10.1371/journal.pone.0035176 (PMC3335102; doi:10.1371/journal.pone.0035176)
Supplement: Table S1 — Primers used for RT-PCR of potato soft-rot pathogens. (DOC) [file pone.0035176.s001.doc]

**Table S1. Primers used for RT-PCR of potato soft-rot pathogens**

| **Strain** | **Gene** | **Primers** |
| --- | --- | --- |
|  |  |  |
| ***P. atrosepticum* CFBP 1526^T^** | *16S rRNA* | 16SF 5' GAGTAATGTCTGGGAAACTGCC 3' |
|  |  | 16SR 5' CTCTACGCATTTCACCGCTAC 3' |
|  | *recA* | RecAF 5' GATGAGAACAAACAAAAGGCACT 3' |
|  |  | RecAR 5' CTCTTTCAGGAAATTACAGGCATT 3' |
|  | *MetK* | MetKFPecto 5' GCGGTGATGGAAGAGATCAT 3' |
|  |  | MetKRPecto 5' ACGGCCAAAGTGACCATAAG 3' |
|  | *luxI* | LuxIFPca 5' CGGTGTAGAGGGTGATCAGG 3' |
|  |  | LuxIRPca 5' GGCCACTCTCGCAACTTACT 3' |
|  | *luxS* | LuxSFPca 5' TTTACCGTTGACCACACTCG 3' |
|  |  | LuxSRPca 5' GCCAGATCGTCATTTTGGTT 3' |
|  |  |  |
| ***P.* *carotovorum* CFBP 2046^T^** | *16S rRNA* | 16SF 5' GAGTAATGTCTGGGAAACTGCC 3' |
|  |  | 16SR 5' CTCTACGCATTTCACCGCTAC 3' |
|  | *recA* | RecAF 5' GATGAGAACAAACAAAAGGCACT 3' |
|  |  | RecAR 5' CTCTTTCAGGAAATTACAGGCATT 3' |
|  | *MetK* | MetKFPecto 5' GCGGTGATGGAAGAGATCAT 3' |
|  |  | MetKRPecto 5' ACGGCCAAAGTGACCATAAG 3' |
|  | *luxI* | LuxIFPcc 5’ GATCGACTGAATTGGGCTGT 3' |
|  |  | LuxIRPcc 5' TCATCAACGGGAAGGAAAAC 3' |
|  | *luxS* | LuxSFPcc 5' GGGTCGCTAAAACCATGAAA 3' |
|  |  | LuxSRPca 5' GCCAGATCGTCATTTTGGTT 3' |
|  |  |  |
| ***D. chrysanthemi* CFBP 2048^T^** | *16S rRNA* | 16SF 5' GAGTAATGTCTGGGAAACTGCC 3' |
|  |  | 16SR 5' CTCTACGCATTTCACCGCTAC 3' |
|  | *recA* | RecAF 5' GATGAGAACAAACAAAAGGCACT 3' |
|  |  | RecAR 5' CTCTTTCAGGAAATTACAGGCATT 3' |
|  | *MetK* | MetKFDck 5' GCCGTGATGGAAGAGATCAT 3' |
|  |  | MetKRDck 5' AGCTGCGGTTTTCTGGTAGA 3' |
|  | *luxI* | LuxIFDck 5' GGGATGGAGTTCGACGAGTA 3' |
|  |  | LuxIRDck 5' TGGAACCAGTAAAGGCAAGG 3' |
|  | *luxS* | LuxSFDck 5' TACTCGTATGGCTGCACCTG 3' |
|  |  | LuxSRDck 5' TTCAACTCAGGGATCTTACG 3' |
|  | *iaaM* | IaaMF 5' CTGTTTATGCTCACGCGAAA 3' |
|  |  | IaaMR 5' CGTCTGCATAGCACCTTCAA 3' |
|  |  |  |
